# The Basophil Activation Test Is the Most Accurate Test in Predicting Allergic Reactions to Baked and Fresh Cow's Milk During Oral Food Challenges

**DOI:** 10.1111/all.16675

**Published:** 2025-08-13

**Authors:** Irene Bartha, Holly Boyd, Ru‐Xin Foong, Marta Krawiec, Andreina Marques‐Mejias, Hannah F. Marshall, Suzana Radulovic, Faye Harrison, Grammatiki Antoneria, Zainab Jama, Matthew Kwok, Ewa Pietraszewicz, Malak Eghleilib, Cristian Ricci, Tom Marrs, Gideon Lack, George Du Toit, Alexandra F. Santos

**Affiliations:** ^1^ Department of Women and Children's Health (Pediatric Allergy), School of Life Course Sciences, Faculty of Life Sciences and Medicine King's College London London UK; ^2^ Children's Allergy Service, Evelina London Children's Hospital Guy's and St Thomas' Hospital London UK; ^3^ Peter Gorer Department of Immunobiology, School of Immunology and Microbial Sciences King's College London London UK; ^4^ Africa Unit for Transdisciplinary Health Research (AUTHeR) North‐West University Potchefstroom South Africa

**Keywords:** anaphylaxis, baked milk, basophil activation test, cow's milk allergy, diagnosis, food allergy, fresh milk, oral food challenges, skin prick test, specific IgE

## Abstract

**Background:**

Cow's milk is the most common cause of food allergy and related fatalities. Consumption of baked milk (BM) has been associated with better prognosis, nutrition and quality‐of‐life.

**Methods:**

The BAT 2 milk study (NCT03309488) was a diagnostic study of cow's milk allergy designed according to STARD guidelines. All children had an oral food challenge (OFC) to BM, and those who tolerated BM were offered an OFC to fresh milk (FM). The diagnostic performance of the basophil activation test (BAT), skin prick test (SPT) and specific IgE (sIgE) was assessed in comparison with OFC.

**Results:**

Out of the 150 children studied, 85% tolerated BM and 56% also tolerated FM. BAT had the best diagnostic accuracy (area under the receiver operator characteristic curve 0.90 for BM and 0.81 for FM) compared with the other tests for both BM (sIgE: 0.79 and SPT: 0.70) and FM (sIgE: 0.74 and SPT: 0.78) allergies. Using 100% sensitivity and 100% specificity cut‐offs to select patients for OFC would have led to the following proportion of children having OFC (and positive OFC) to BM: 99% (13%) for SPT to baked milk, 82% (17%) for sIgE to boiled milk, and 49% (24%) for BAT to baked milk. In children younger than 2 years, only 27% required an OFC following BAT to BM (compared to 100% and 81% following SPT or sIgE to milk extract, respectively), with 100% diagnostic accuracy.

**Conclusions:**

BAT was the best test to identify children who reacted on OFC to BM or FM. Using 100% sensitivity and 100% specificity cut‐offs, BAT ensured the lowest need for OFC and 100% diagnostic accuracy.

**Trial Registration:**

clinicaltrials.gov identifier: NCT03309488

## Graphical abstract

The BAT had larger areas under the receiver‐operator‐characteristic curve to identify BM (0.90) and FM (0.81) allergies compared to SPT (0.70 and 0.78) and sIgE (0.79 and 0.74) to cow's milk. Using optimal cut‐offs for BM allergy, sensitivity and specificity were 80/90% for BAT, 70/64% for SPT, and 90/66% for sIgE to cow's milk. Using 100% sensitivity and 100% specificity cut‐offs followed by OFC to BM led to 100% diagnostic accuracy, with BAT requiring the lowest number of OFC (49%, 24% positive) compared to SPT (100%, 14% positive) and sIgE (88%, 16% positive). BAT, basophil activation test; BM, baked milk; FM, fresh milk. OFC, oral food challenge; SPT, skin prick test; sIgE, specific IgE.

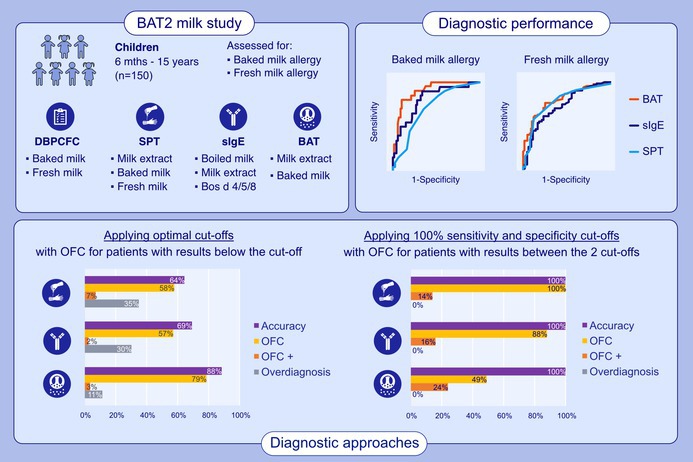

## 1 Introduction

Cow's milk allergy (CMA) is one of the most common food allergies worldwide, with a recently estimated prevalence of 5.7% (95% confidence interval 4.4–6.9) [1]. Being a staple food, accidental reactions to cow's milk are common [2]. CMA is, together with nut allergies, the most common cause for food anaphylaxis fatalities, particularly in teenagers and young adults [3, 4, 5]. Although most affected children resolve their CMA spontaneously, CMA can be persistent throughout life [6]. The ability to tolerate baked milk (BM) and its regular consumption have been associated with a better prognosis of CMA and a better quality of life [7]. However, currently used tests, such as the skin prick test (SPT) and specific IgE (sIgE) do not enable accurate identification of children who can tolerate BM without doing an oral food challenge (OFC) [8]. Access to OFC is limited due to the increasing number of patients being referred and to the time‐consuming, resource‐intensive, and risky nature of this procedure [9]. Additionally, while waiting for OFC, patients need to avoid the allergen, and this can increase the risk of food allergy, especially in young children. Some children and families have significant anxiety related to the idea of being exposed to their food allergen, particularly older children and teenagers, who are used to avoiding the food and may also have developed food aversion over time.

A precise diagnosis of CMA is extremely important to prevent potentially severe allergic reactions and to allow timely introduction of the food in the child's diet. The recently published EAACI guidelines for the diagnosis of IgE‐mediated food allergy [10] recommend a stepwise approach to food allergy diagnosis, with the first steps being history and SPT or sIgE to allergen extracts and subsequent steps including sIgE to individual allergens and the basophil activation test (BAT) before patients are referred for OFCs. However, for CMA, sIgE to individual allergens was not found to be more informative than sIgE to cow's milk extract, and there were not enough studies on BAT to milk to allow for meta‐analyses and formal inclusion of BAT as recommended tests for CMA diagnosis [8].

In the BAT2 milk study, we aimed to determine the diagnostic accuracy of BAT in the diagnosis of BM and fresh milk (FM) allergies, and to compare it with the diagnostic accuracy of tests currently used in clinical practice, such as SPT and sIgE. Children were referred by various clinicians, and all participants underwent OFCs, double‐blind placebo‐controlled food challenges (DBPCFC) if 12 months of age or older and open OFCs for infants (i.e., younger than 12 months). The diagnostic performance of all tests was determined by comparison with the reference standard, OFC.

## 2 Methods

### 2.1 Study Design

This diagnostic study was designed as per Standards for Reporting of Diagnostic Accuracy Studies (STARD) guidelines [11] and registered at clinicaltrials.gov with NCT03309488. The primary outcome of the BAT2 study was the diagnostic accuracy of BAT. The added value of BAT to other tests constituted secondary outcomes.

All participants attended for BM OFC. Children who tolerated BM had a second OFC to FM. They had SPT, blood collection for serology and BAT, and OFC to BM on the same day. If the OFC to FM occurred 6 months or more after the OFC to BM, SPT and blood collection for serology and BAT were repeated. The diagnostic accuracy of BAT, SPT and sIgE was determined in comparison with the reference standard OFC.

The study was approved by the London–Westminster Research Ethics Committee (ref 17/LO/0296) and the UK Health Research Authority. Informed consent was obtained from a parent or guardian, and assent was obtained from the child before any study procedures.

### 2.2 Eligibility Criteria

Children aged 6 months to 15 years needing an OFC to cow's milk were prospectively and consecutively invited to take part in the BAT2 study by a variety of clinicians from specialized Pediatric Allergy clinics in London. Eligible children either had: (1) a history of an immediate‐type allergic reaction to cow's milk or (2) no history of cow's milk consumption and/or (3) evidence of IgE sensitization as documented by SPT and/or serum sIgE. Exclusion criteria were: clinically significant chronic illness other than atopic diseases; previous history of severe life‐threatening reaction to the suspected food with documented decrease in oxygen saturation (< 90%), hypotension (≥ 20% reduction in systolic blood pressure) and/or admission to intensive care; unwillingness to comply with study procedures, namely to undergo a diagnostic food challenge; contraindication for diagnostic food challenge (such as uncontrolled atopic diseases, chronic medical conditions that pose significant risk in the event of anaphylaxis or treatment of anaphylaxis, inability to discontinue medications that might interfere with assessment or safety, treatment within 7–14 days with systemic steroids or prolonged high‐dose systemic steroids or immunosuppressants); current treatment with omalizumab, food allergen immunotherapy, or other systemic immunomodulatory treatments; and inability to stop antihistamines prior to SPT.

### 2.3 Skin Prick Testing

SPT was done using cow's milk extract (ALK Abello, Madrid, Spain), FM and BM slurry (prepared on the day of the OFC with 1 g of the challenge food in 10 mL of saline) and a single‐headed metal lancet. Solutions of 10 mg/mL histamine dihydrochloride and 50% glycerol in buffered saline were used as positive and negative controls, respectively. The size of the wheal was determined as the arithmetic average of two perpendicular diameters, including the longest one, and recorded after 15 min.

### 2.4 IgE Testing

The levels of total IgE, sIgE to cow's milk, boiled milk, alpha‐lactalbumin (Bos d 4), beta‐lactoglobulin (Bos d 5) and casein (Bos d 8) were determined using the standardized immunoenzymatic assay ImmunoCAP (Thermo fisher, Uppsala, Sweden).

### 2.5 Basophil Activation Testing

BAT was performed in the Santos Lab at King's College London, as previously described [12]. Whole blood collected in lithium heparin (100 μL per condition) was incubated with an equal volume of milk extract (ALK‐Abello), BM (Sigma‐Aldrich, Poole, UK), anti‐IgE (1 μg/mL, Sigma‐Aldrich, Poole, United Kingdom), formyl‐methionyl‐leucyl‐phenylalanine (fMLP, 1 μM, Sigma‐Aldrich), diluted in RPMI (GIBCO, Paisley, United Kingdom), or RPMI alone. BM was prepared as a single batch by heating in an oven at 180°C for 20 min and stored at −80°C thereafter. After incubation at 37°C for 30 min, samples were placed on ice and cold EDTA was added to stop degranulation. Staining with CD123‐FITC, CD203c‐PE, HLA‐DR‐PerCP and CD63‐APC (all Biolegend, San Diego, Calif) using individual tubes with pre‐mixed lyophilized antibodies customized for the Santos Lab, was followed by red blood cell lysis using Pharmlyse (BD Biosciences, San Diego, CA). Flow cytometry was performed using FACS Fortessa with FACSDiva software (BD Biosciences, San Jose, Calif) and analyzed using FlowJo software (version 7.6.1; TreeStar, Ashland, Ore). Basophils were gated as SSC^low^/CD203c+/CD123+/HLA‐DR‐ [13]. Basophil activation was expressed as %CD63+ basophils and SI CD203c. Non‐responder basophils were defined as %CD63+ basophils below 5% to anti‐IgE and allergen.

### 2.6 Oral Food Challenges

All children aged 12 months or older had DBPCFC. Open incremental OFC was done for children younger than 12 months of age to improve feasibility as open OFC requires fewer doses and smaller volumes of food. Table S1 shows the dose regimens for OFC to BM and FM for different age groups. The outcome of the OFC was determined according to the Practall guidelines [14].

### 2.7 Statistical Analyses

Categorial variables were compared with Chi‐Square or Fisher's Exact Test, as appropriate, whereas continuous variables were compared with Mann–Whitney *U* Test between groups. Receiver operator characteristic (ROC) curve analyses were performed to assess the diagnostic accuracy of the various tests. Simulation and resampling techniques were conducted to assess internal validity. Optimal cut‐offs were determined by the Youden index, compared with the outcome of OFC. Positive and negative cut‐offs were defined by the highest point in the ROC curve with 100% sensitivity and the lowest point in the same curve with 100% specificity. All statistical evaluations were performed by SPSS version 27. Internal validation was performed by numerical computation and bootstrap resampling performed using the R software. Statistical tests were two‐tailed and type‐I error rate was set to 5% (*α* = 0.05).

## 3 Results

### 3.1 Study Cohort

**FIGURE 1:**
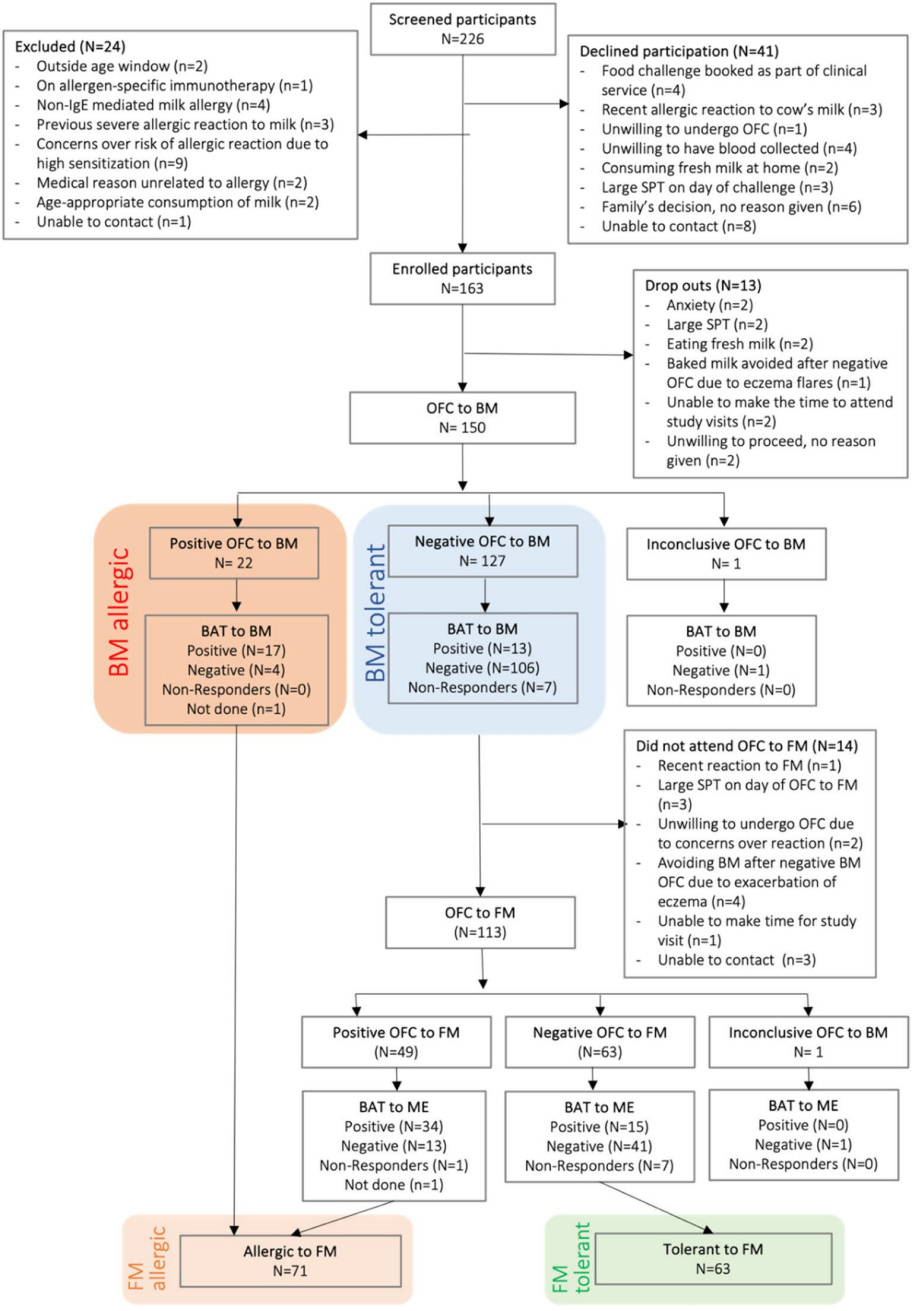
Flow diagram from the BAT2 cow's milk study assessing the utility of the basophil activation test in predicting clinical reactivity to baked milk and fresh milk during DBPCFC, built according to the STARD guidelines for diagnostic studies [11]. BAT, basophil activation test; BM, baked milk; DBPCFC, double‐blind placebo‐controlled food challenge; FM, fresh milk.

Children requiring an OFC to cow's milk were referred to the study by a variety of clinicians and were screened by a member of the BAT2 study team to confirm eligibility. During this process, 78 children were excluded: 24 did not meet selection criteria, 41 declined participation, and 13 did not complete their baseline visit. Figure 1 provides more detail on the underlying reasons. Table S2 indicates the proportion of children meeting each inclusion criterion and their OFC outcomes. Out of the 150 children who completed their baseline visit and their BM OFC, 127 did not react to BM and were offered a FM OFC, which 113 completed. There were two inconclusive OFC, one to BM and one to FM. In the end, 149 children had complete assessment for BM allergy (22 allergic and 127 tolerant) and 134 for FM allergy (71 allergic and 63 tolerant). Table 1 presents the demographic, clinical, and immunological characteristics of study participants, divided according to whether they were allergic or tolerant to BM and FM. Table S3 reports the clinical characteristics of positive OFC.

**TABLE 1:** Patient demographic, clinical and immunological characteristics according to their allergic status to baked milk (*n* = 149) and fresh milk (*n* = 134) as determined by oral food challenges.

Patient characteristics	Baked milk allergic (*n* = 22)	Baked milk tolerant (*n* = 127)	*p*	Fresh milk allergic (*n* = 71)	Fresh milk tolerant (*n* = 63)	*p*
Age (years)	4.2 (2.4; 6.4)	4.0 (2.3; 7.6)	0.911	4.2 (2.4; 7.6)	4.4 (2.7; 9.0)	0.861
Gender (% females)	7 (32%)	55 (43%)	0.357	31 (44%)	26 (41%)	0.699
Ethnicity						
White	12 (55%)	72 (57%)	0.436	42 (59%)	36 (57%)	
Black	3 (14%)	11 (9%)		4 (6%)	9 (14%)	
Asian	3 (14%)	6 (5%)		8 (11%)	0 (0%)	**0.037**
Chinese	0 (0%)	9 (7%)		5 (7%)	2 (3%)	
Mixed	3 (14%)	24 (19%)		10 (14%)	13 (21%)	
Other	1 (5%)	5 (4%)		2 (3%)	3 (5%)	
History of allergic reaction to milk (%)						
Baked milk	7 (32%)	31 (24%)	0.440	24 (34%)	11 (18%)	0.048
Fresh milk	17 (77%)	97 (76%)	1.0	56 (79%)	47 (75%)	0.682
Atopic eczema (%)	19 (86%)	100 (79%)	0.568	53 (75%)	54 (86%)	0.133
Other food allergies (%)	20 (91%)	2 (9%)	0.090	67 (94%)	4 (5%)	0.534
Allergic rhinitis (%)	6 (27%)	45 (35%)	0.627	22 (31%)	24 (38%)	0.467
Asthma (%)	5 (23%)	26 (21%)	0.781	17 (24%)	10 (16%)	0.285
SPT to cow's milk extract (mm)	4 (3; 5)	3 (0; 4)	**0.006**	4 (3; 5)	1 (0; 3)	**< 0.001**
SPT to fresh milk (mm)	8 (5; 9)	5 (3; 8)	**0.014**	7 (5; 9)	4 (0; 5)	**< 0.001**
SPT to baked milk slurry (mm)	5 (3; 6)	1 (0; 3)	**< 0.001**	—	—	—
Difference CME and FM SPT	3 (2; 5)	3 (1; 5)	0.403	3.5 (1.5; 5.0)	1.5 (0; 3.0)	**< 0.001**
Ratio CME/FM SPT	0.56 (0.39; 0.75)	0.50 (0.27; 0.67)	0.185	0.53 (0.37; 0.69)	0.47 (0.25; 0.75)	0.395
Specific IgE to Boiled Milk (kU_A_/L)	5.53 (1.14; 14.18)	0.31 (0.07; 1.44)	**< 0.001**	1.21 (0.27; 5.42)	0.16 (0.03; 0.51)	**< 0.001**
Specific IgE to Cow's milk (kU_A_/L)	6.29 (1.47; 14.73)	0.48 (0.18; 1.98)	**< 0.001**	1.55 (0.41; 8.26)	0.35 (0.12; 0.74)	**< 0.001**
Specific IgE to Bos d 4 (kU_A_/L)	0.70 (0.09; 3.57)	0.11 (0.02; 0.83)	**0.042**	0.33 (0.02; 2.89)	0.07 (0.01; 0.17)	**< 0.001**
Specific IgE to Bos d 5 (kU_A_/L)	0.62 (0.05; 2.38)	0.15 (0.06; 0.61)	0.247	0.28 (0.08; 0.,82)	0.13 (0.03; 0.36)	**0.004**
Specific IgE to Bos d 8 (kU_A_/L)	3.09 (1.12; 13.15)	0.16 (0.03; 0.85)	**< 0.001**	0.68 (0.14; 3.30)	0.11 (0.01; 0.28)	**< 0.001**
BAT to milk extract at 100ng/mL (%CD63+ Basophils)	24.99 (16.70; 36.28)	2.58 (0.61; 8.94)	**< 0.001**	9.45 (2.80; 28.72)	1.17 (0.16; 3.65)	**< 0.001**
BAT to milk extract at 100ng/mL (SI CD203c)	3.48 (2.58; 4.66)	1.22 (1.04; 1.86)	**< 0.001**	1.97 (1.30; 3.54)	1.07 (1.0; 1.30)	**< 0.001**
BAT to baked milk at 100ng/mL (%CD63+ Basophils)	18.01 (9.43; 29.24)	0.85 (0; 3.42)	**< 0.001**	4.69 (0.84; 20.41)	0.20 (0; 1.32)	**< 0.001**
BAT to baked milk at 100ng/mL (SI CD203c)	3.54 (2.06; 4.54)	1.09 (1.00; 1.35)	**< 0.001**	1.51 (1.13; 3.55)	1.04 (0.97; 1.11)	**< 0.001**

*Note:* Statistical significance was indicated in bold for *P* values < 0.05.

Abbreviations: BAT, basophil activation test; CME, cows milk extract; FM, fresh milk; SPT, skin prick test.

### 3.2 The Basophil Activation Test Is Superior to Other Tests in Identifying Children Who Can Tolerate Baked Milk

Eighty‐five percent of study participants tolerated BM, and 56% also tolerated FM. Figure S1 shows the proportion of children passing OFC to BM and FM in different age groups. The results of SPT, sIgE and BAT were statistically significantly higher in BM allergic children compared to BM tolerant children, except for sIgE to beta‐lactoglobulin (Bos d 5) in BM allergy (Table 1). The difference between SPT to FM and milk extract and the ratio between SPT to milk extract and SPT to FM did not distinguish these two groups. The SPT difference between FM and milk extract was statistically significantly different only between FM allergic and FM tolerant groups.

**FIGURE 2:**
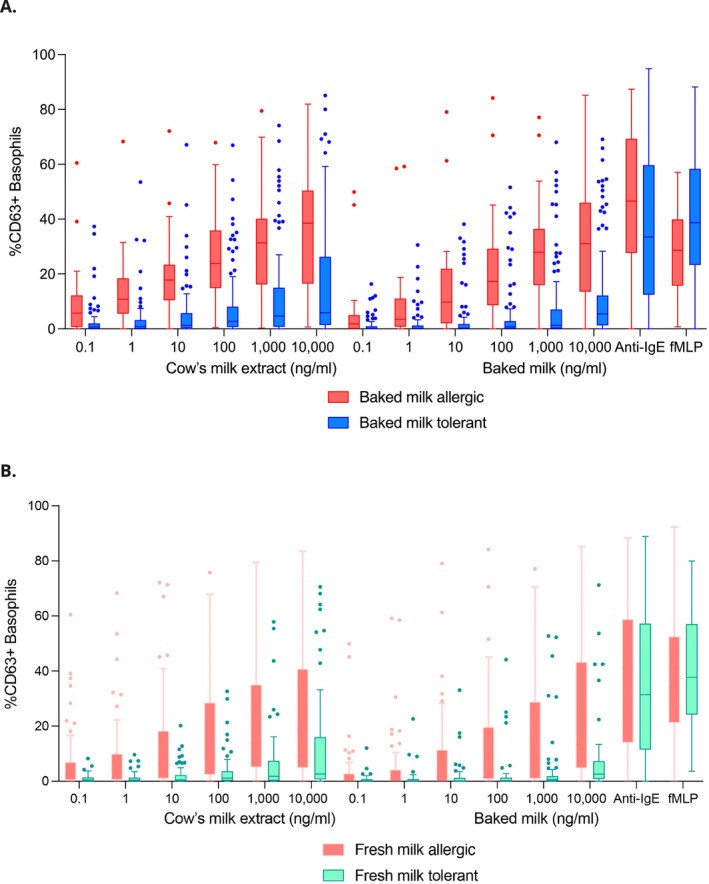
Results of basophil activation test in children allergic to and tolerant of baked milk (A) and fresh milk (B) during oral food challenges.

The BAT provides a clear distinction between allergic and tolerant children to either BM or FM (Figure 2). Only 5% (7/149) of participants had non‐responder basophils, and they all were BM tolerant, and all except 1 were tolerant to FM. Figure S2 shows the same data with patients grouped according to allergic status to both BM and FM (i.e., (1) allergic to all forms of milk, (2) allergic to FM but tolerant to BM and (3) tolerant to all forms of milk), evidencing that BAT discriminates well among the 3 phenotypes of milk allergy. Some milk tolerant patients had a positive BAT for both BM and FM allergies, which was uncommonly observed in peanut allergy (Table S4) [15].

### 3.3 Diagnostic Cut‐Offs for the Basophil Activation Test and Other Tests

**FIGURE 3:**
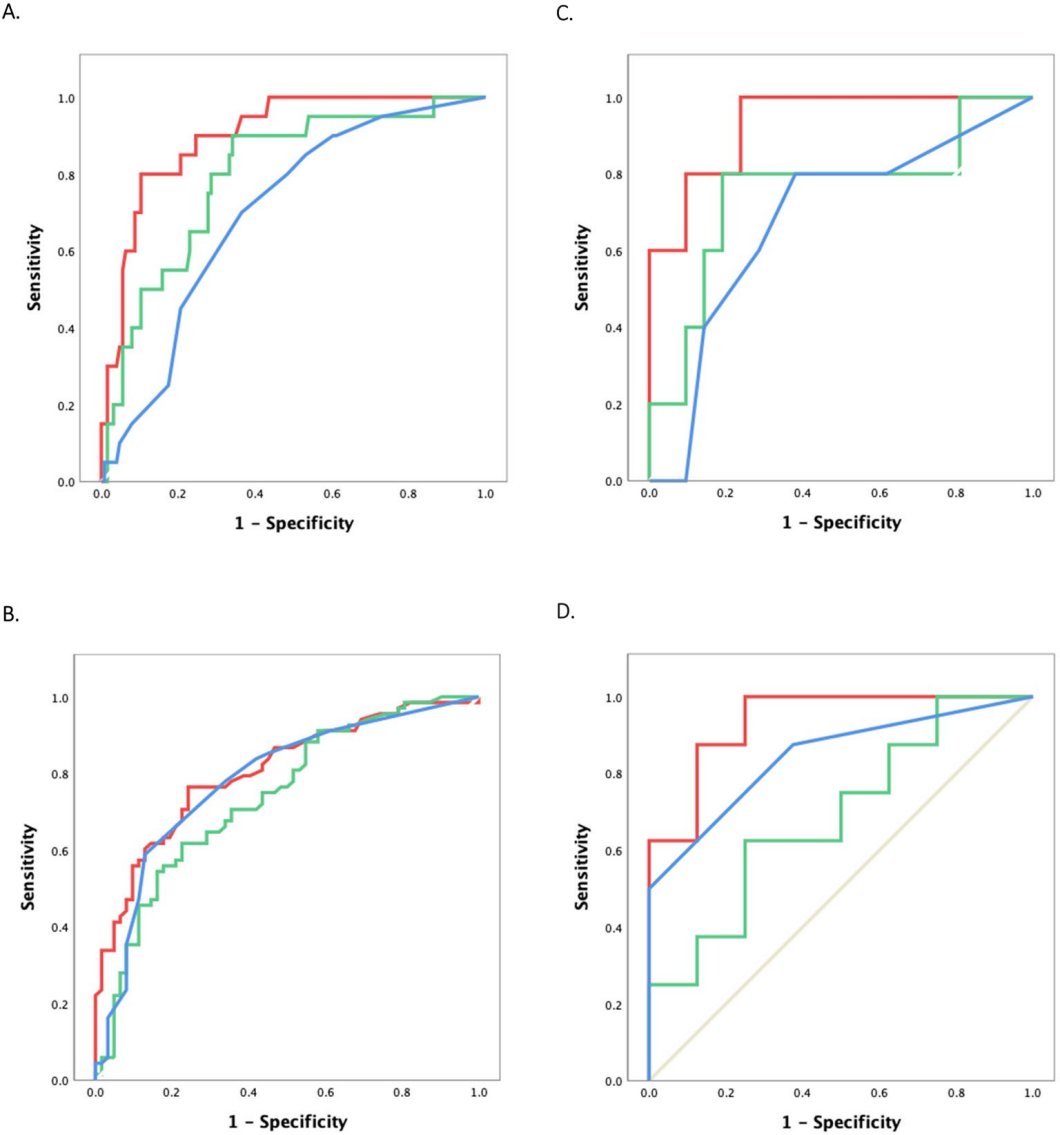
Receiver Operator Characteristic curves for the best tests for each test modality (BAT in red, specific IgE in green and SPT in blue). BAT, basophil activation test; SPT, skin prick test; sIgE, specific immunoglobulin E. (A). Baked milk allergy in children of all ages. (B). Fresh milk allergy in children of all ages. (C) Baked milk allergy in children younger than 2 years. (D) Fresh milk allergy in children younger than 2 years.

ROC curve analyses confirmed the superiority of BAT compared with sIgE and SPT to identify children who reacted during OFC to BM (Figure 3A) or during OFC to FM (Figure 3C). Figure S3 shows ROC curves for the different allergen preparations used for SPT, specific IgE, and BAT. The most discriminative milk extract concentration for BAT was 100 ng/mL for both BM and FM allergies. The basophil activation marker CD203c offered a slightly superior distinction with higher specificity compared to CD63 [15]. Table 2A and C show the diagnostic cut‐offs identified, including optimal cut‐offs (defined by the Youden index as the one with the best balance between sensitivity and specificity), cut‐offs with 100% sensitivity (useful to exclude allergy) and cut‐offs with 100% specificity (useful to confirm allergy).

For BM allergy, the optimal cut‐off for BAT was 11.1% CD63+ basophils or SI CD203c of 1.88, with sensitivity/specificity of 91%/78% and 80%/90%, respectively. For children younger than 2 years, the optimal cut‐off for BM allergy (which coincided with the 100% sensitivity cut‐off) was SI CD203c of 1.19 with 100% sensitivity and 76% specificity. For FM allergy, the optimal cut‐off for BAT was 3.57% CD63+ basophils or SI CD203c of 1.29, with sensitivity/specificity of 71%/76% and 77%/76%, respectively. For children younger than 2 years, BAT's optimal cut‐off for FM allergy was SI CD203c of 0.88 with 88% sensitivity and 88% specificity.

### 3.4 The Basophil Activation Test Is Particularly Useful in Children Younger Than 2 Years of Age

The performance of BAT was particularly more accurate than the other tests in the age group below 2 years old, especially for FM allergy (Figures 2B and 3D). Figure S4 illustrates high basophil activation in allergic children, both younger and older, whereas sIgE tends to be lower in allergic children in their early years. For diagnostic cut‐offs for this age group, see Table 2B,D.

**TABLE 2:** Diagnostic cut‐offs (optimal, 100% sensitivity and 100% specificity) for the basophil activation test and the other tests defined based on the outcome of oral food challenges.

Diagnostic tests	Cut‐off		AUC ROC	Sensitivity	Specificity	PPV	NPV	Diagnostic accuracy	TP/FP	TN/FN
(A) Baked milk allergy in all children										
SPT to milk extract	100% S	0 mm	0.50	100%	0%	14%	—*	14%	20/126	0/0
	OPTIMAL	3 mm	0.67	70%	64%	23%	93%	64%	14/46	80/6
	100% Sp	11 mm	0.50	0%	100%	—*	86%	86%	0/0	126/20
SPT to baked milk	100% S	0 mm	0.50	100%	0%	14%	—*	14%	20/126	0/0
	OPTIMAL	3 mm	0.78	80%	25%	34%	96%	76%	16/31	95/4
	100% Sp	9 mm	0.53	5%	100%	100%	87%	87%	1/0	126/19
SIgE to cow's milk (KU/L)	100% S	0.11	0.57	100%	14%	16%	100%	25%	20/109	17/0
	OPTIMAL	0.85	0.78	90%	66%	30%	98%	69%	18/43	83/2
	100% Sp	58.7	0.50	0%	100%	—*	86%	86%	0/0	126/20
SIgE to boiled milk (KU/L)	100% S	0.05	0.61	100%	21%	17%	100%	32%	20/99	27/0
	OPTIMAL	0.86	0.80	90%	71%	33%	98%	73%	18/37	89/2
	100% Sp	68.7	0.50	0%	100%	—*	86%	86%	0/0	126/20
BAT to milk extract (%CD63 100 ng/mL)	100% S	0.7	0.64	100%	27%	18%	100%	37%	20/92	34/0
	OPTIMAL	11.1	0.84	91%	78%	39%	98%	79%	18/98	28/2
	100% Sp	67.4	0.52	5%	100%	100%	87%	87%	1/0	126/19
BAT to baked milk (SI CD203c 100 ng/mL)	100% S	1.13	0.78	100%	56%	27%	100%	62%	20/55	71/0
	OPTIMAL	1.88	0.85	80%	90%	55%	97%	88%	16/13	113/4
	100% Sp	6.23	0.58	15%	100%	100%	88%	88%	3/0	126/17
(B) Baked milk allergy in children younger than 2 years										
SPT to milk extract (mm)	100% S	0 mm	0.50	100%	0%	19%	—*	19%	5/21	0/0
	OPTIMAL	3 mm	0.71	80%	62%	33%	93%	65%	4/8	13/1
	100% Sp	8 mm	0.50	0%	100%	—*	81%	81%	0/0	21/5
SIgE to cow's milk (KU/L)	100% S	0.11	0.60	100%	19%	23%	100%	35%	5/17	4/0
	OPTIMAL	0.78	0.81	80%	81%	50%	94%	81%	4/4	17/1
	100% Sp	27.35	0.60	20%	100%	100%	84%	81%	1/0	21/4
BAT to baked milk (SI CD203c at 100 ng/mL)	100% S	1.19	0.88	100%	76%	50%	100%	81%	5/5	16/0
	OPTIMAL									
	100% Sp	1.87	0.80	60%	100%	100%	91%	92%	3/0	21/2
(C) Fresh milk allergy in all children										
SPT to milk extract	100% S	0 mm	0.50	100%	0%	52%	—*	52%	68/62	0/0
	OPTIMAL	3 mm	0.73	59%	87%	83%	66%	72%	40/8	54/28
	100% Sp	8 mm	0.52	4%	100%	100%	49%	50%	3/0	62/65
SIgE to cow's milk (KU/L)	100% S	0.07	0.55	100%	10%	55%	100%	57%	68/56	6/0
	OPTIMAL	0.77	0.70	62%	77%	75%	65%	69%	42/14	48/26
	100% Sp	49.2	0.51	2%	100%	100%	48%	48%	1/0	62/67
BAT to milk extract (%CD63 at 100 ng/mL)	100% S	0	0.59	100%	18%	57%	100%	61%	68/51	11/0
	OPTIMAL	3.57	0.73	71%	76%	76%	70%	73%	48/15	47/20
	100% Sp	33.72	0.60	19%	100%	100%	53%	58%	13/0	62/55
BAT to milk extract (SI CD203c at 100 ng/mL)	100% S	0	0.50	100%	0%	52%	—*	52%	68/62	0/0
	OPTIMAL	1.29	0.76	77%	76%	78%	75%	76%	52/15	47/16
	100% Sp	3.71	0.61	22%	100%	100%	54%	59%	15/0	62/53
(D) Fresh milk allergy in children younger than 2 years										
SPT to milk extract (mm)	100% S	0 mm	0.50	100%	0%	50%	—*	50%	8/8	0/0
	OPTIMAL	3 mm	0.75	75%	75%	75%	75%	75%	6/2	6/2
	100% Sp	4 mm	0.75	50%	100%	100%	67%	75%	4/0	8/4
SIgE to cow's milk (KU/L)	100% S	0.09	0.63	100%	25%	57%	100%	63%	8/6	2/0
	OPTIMAL	0.24	0.69	63%	75%	71%	67%	69%	5/2	6/3
	100% Sp	1.92	0.63	25%	100%	100%	57%	63%	2/0	8/6
BAT to milk extract (%CD63 at 100 ng/mL)	100% S	0.25	0.88	100%	75%	80%	100%	88%	8/2	6/0
	OPTIMAL	0.88	0.81	88%	88%	88%	88%	88%	7/1	7/1
	100% Sp	4.07	0.81	63%	100%	100%	73%	81%	5/0	8/3

Abbreviations: AUC ROC, area under the receiver operating characteristic curve; BAT,basophil activation test; NPV, negative predictive value; PPV, positive predictive value; S, sensitivity; sIgE, specific immunoglobulin E; Sp, specificity; SPT,skin prick test; TN/FN, true negative/false negative; TP/FP, truepositive/false positive.

* Not possible to calculate due to divisor being zero.

### 3.5 Integrating the Basophil Activation Test in the Diagnostic Work‐Up of Milk Allergy Can Improve Patients' Management and Outcomes

With superior diagnostic accuracy, compared to sIgE and SPT, BAT could enable the identification of children who can safely tolerate BM. Applying optimal cut‐offs, BAT had the highest diagnostic accuracy when compared to the outcome of BM OFC: 88% for BAT to BM (79% for BAT to milk extract) whereas this was 76% for SPT to BM (64% for SPT to milk extract), 73% for specific IgE to boiled milk (69% for specific IgE to cow's milk). Interestingly, often, the diagnostic accuracy of all tests was higher when applying the 100% specificity cut‐offs as a single cut‐off compared to applying the optimal cut‐off. However, if 100% specificity cut‐offs were used to decide about OFC referral, a higher proportion of positive OFC would be expected (Table 2).

Applying 100% sensitivity and 100% specificity cut‐offs to identify tolerant and allergic patients, respectively, and doing OFC in patients with results between these 2 cut‐offs would result in 100% diagnostic accuracy. Following this approach, BAT was the test that would have required the lowest number of OFC (Table 3). For instance, the 100% sensitivity cut‐off for BAT (e.g., SI CD203c of 1.13 following stimulation with BM) identified 71 out of 149 children as able to tolerate BM based on BAT, without doing OFC, with 0 false negatives. Three patients had BAT results above the 100% specificity cut‐off, confirming BM allergy. Out of the remaining patients, 72 (49% of the cohort) would undergo OFC and 24% would react. This means that, in clinical practice, using BAT as a single test would have reduced the need for OFC by 51%, with a 24% chance of a positive OFC for those who did require this, and an overall 100% diagnostic accuracy.

**TABLE 3:** Proportion of children requiring oral food challenges to baked milk, and experiencing positive oral food challenges, using positive and negative cut‐offs for individual tests. Positive (i.e., 100% specificity) cut‐offs were used to confirm allergy, negative (i.e., 100% sensitivity) cut‐offs to exclude allergy, and patients with results between cut‐offs would need an OFC.

Age group	TESTS	Tolerant	Equivocal	Allergic	OFC‐	OFC+	%OFC	% OFC+
All ages	SPT ME	0	146	0	126	20	100%	14%
	SPT BM	0	145	1	126	19	99%	13%
	sIgE Cow's Milk	17	129	0	109	20	88%	16%
	sIgE Boiled Milk	27	119	0	99	20	82%	17%
	BAT milk extract	34	111	1	92	19	76%	17%
	BAT Baked milk	71	72	3	55	17	49%	24%
< 2 years	SPT Milk Extract	0	26	0	21	5	100%	19%
	sIgE Cow's Milk	4	21	1	17	4	81%	19%
	BAT Baked Milk	16	7	3	5	2	27%	29%

Abbreviations: BAT, basophil activation test; BM, baked milk; ME, milk extract; OFC, oral food challenge; sIgE, specific immunoglobulin E; SPT, skin prick test.

In children younger than 2 years, BAT was particularly useful to identify children who tolerated baked milk, compared to the other tests: the diagnostic accuracy of BAT was 81% and 92% for optimal and 100% specificity cut‐offs, compared to 81%/81% for specific IgE to cow's milk and 65% and 81% for SPT to milk extract. BAT allowed a 73% reduction in OFC, compared with 0% for SPT to milk extract and 19% for sIgE to cow's milk.

The same diagnostic approaches could be used for FM allergy. However, the performance of all tests was less good for FM allergy compared to BM allergy, probably because of the time gap between testing and the OFC, which was longer for FM than for BM (median and interquartile range of 42 (13; 81) days and 0 (0; 0) days, respectively).

## 4 Discussion

Cow's milk allergy is the most common food allergy worldwide. Cow's milk is the food most implicated in anaphylaxis fatalities, alongside nuts [3, 4]. Consumption of BM has been associated with a better prognosis in terms of likelihood of resolution of milk allergy and better quality of life. Most milk allergic children tolerate BM; however, currently used allergy tests are poor predictors of safe BM introduction and OFC is ultimately required to confirm tolerance [16]. Given the limited capacity for OFC, the long waiting lists lead to delayed reintroduction of milk in the diet with an increased chance of persistent milk allergy [9]. In the BAT2 milk study, we have challenged 150 children to milk and assessed the diagnostic performance of currently used tests and the BAT compared with the reference standard OFC. 85% of these children tolerated BM. BAT had high accuracy and was the best test in identifying children able to tolerate BM in the diet.

The BAT2 milk study is the largest diagnostic study of milk allergy to date, designed according to the STARD guidelines, using the most rigorous methodology, with all patients being submitted to OFC, namely DBPCFC (only infants had open OFC) [11, 14]. We designed OFC with cumulative doses appropriate to different age groups to ensure that the cumulative dose tolerated during the OFC would enable patients to be safe consuming baked goods in the community, particularly of off the shelf produtcs and by patients who were still reactive to FM. Only 2 BM allergic patients reacted to more than 2 g of milk protein during the OFC to BM, and none of these experienced severe symptoms or required adrenaline as part of their treatment. This suggests that 2 g of milk protein is likely to be an appropriate cumulative dose for OFC to BM in all age groups. The cumulative dose of FM tolerated was more variable.

In this study, we observed a lower rate of non‐responders than in other studies [12, 15], which is positive, as non‐responders are a possible limitation of the BAT. We have observed more false positives for BAT to milk than for BAT to peanut [15, 17]. This could possibly be due to masking or modification of cow's milk allergen epitopes by other ingredients of the challenge food—the so‐called matrix effect, which can reduce the allergenicity of milk and lead to a negative OFC (despite positive tests done with the milk allergens alone). The diagnostic performance of BAT depends on the method used to perform the test. We have demonstrated this previously for peanut allergy in a head‐to‐head comparison of 2 different BAT methods, where our in‐house method predicted the OFC outcome correctly in all cases and a commercially available test misdiagnosed 12.5% of cases [13]. Diagnostic cut‐offs can also vary with different BAT methods and may need to be adjusted when using different BAT methodology.

The BAT was the best test to discriminate between BM allergic and tolerant children. The performance of all tests had lower accuracy for FM allergy than for BM allergy, possibly due to the time gap between BM and FM challenges. The interval between blood collection and the FM OFC was never more than 6 months, and children who passed their BM challenge avoided BM during 2 days before the OFC to FM. Although milk allergens were not in circulation when they had their FM challenge, it is possible that continued milk consumption between the BM OFC and the FM OFC changed their allergic status over time. For this reason, the performance of the BAT to milk for FM allergy is probably better than we were able to determine in the BAT2 study—this will be investigated further in the ongoing multicentre BAT Impact study (NCT05309772).

Overall, BAT had the highest diagnostic accuracy for both BM and FM allergies. Even if sometimes the difference in diagnostic accuracy of BAT and other tests was relatively small, we consider that every additional patient diagnosed correctly is clinically relevant. Whether BAT brings additional value to existing tests is a wider question that needs to consider other factors, such as availability of tests, access and capacity to do OFC, acceptable risk and patient preferences. Interestingly, the second‐best test was different for BM allergy and for FM allergy. For BM allergy, sIgE was better than SPT. For FM allergy, it was the reverse. One possible explanation for this could be the longer time gap between test and OFC for FM allergy and the fact that IgE on skin mast cells has a longer half‐life than IgE in the circulation. The fact that children did not have blood collection on the day of each OFC is a limitation of the study; however, the protocol was designed this way to respond to our patient population's perspective—for most families, multiple blood collections would have been a barrier that could have refrained many from agreeing to take part in the study. Another limitation was the fact that placebo doses were interspersed in the middle of active doses. The 1‐day OFC protocol was decided based on previous experience of the LEAP [18, 19] and EAT [18] studies and also the feedback from our patients that a 2‐day OFC would have been unacceptable for many due to requiring travelling and days off work and school. However, two children had anaphylaxis after a placebo dose, which could have been considered ‘placebo reactors’ but given that the active doses had been taken before the placebo dose and given the young age of the children, the objective manifestations and adrenaline being required as part of the treatment, the clinical study team decided that these were unequivocally positive OFC. This decision was supported by the trial data and safety monitoring committee.

The relatively stable chance of tolerance to BM and FM across the age groups was probably due to the cross‐sectional design of the study, to which participants were referred when an OFC was considered indicated by their clinician. An important point to highlight is that the diagnostic performance reported here was generated in this specific cohort of patients. We consider this cohort to be representative of the patient population that we see in our specialized clinic, since children were referred to the study by many different clinicians, and *representative* of the patients who are considered to need an OFC and, therefore, are the population of interest when it comes to the need for additional tests, such as BAT. However, our findings need to be validated in other patient populations, which we are in the process of doing as part of the UK multicenter BAT Impact trial (NCT05309772).

Our study is consistent with previous findings from Novak‐Wegrzyn et al. [20] that children with milk allergy tolerating BM showed an intermediate phenotype in terms of basophil activation. However, our study went further to assess the diagnostic accuracy of BAT and other tests. We defined BAT as the best test to identify children who can tolerate BM. Recent meta‐analyses [8] demonstrated the superiority of sIgE over SPT to cow's milk, which is consistent with our findings for BM allergy (but not for FM allergy). This systematic review of the literature [8] only included one study of BAT to milk and thus could not perform a meta‐analysis for the BAT, due to the insufficient number of studies [21]. Older studies [22, 23] were not captured in the pre‐defined data range of the systematic review and did not look at BM allergy. The BAT to milk study [21] included in the systematic review enrolled a heterogeneous group of patients, including both IgE and non‐IgE mediated CMA, and only 41% of patients were IgE sensitized to milk. Of note, given the underlying immune mechanisms, BAT can be used as a diagnostic test for IgE, but not for non‐IgE mediated CMA.

In clinical practice, given the limited resources and aim to avoid severe reactions, it is common to adopt an optimal cut‐off for allergy tests, above which patients are considered allergic and below which patients are referred for OFC, but this involves a certain degree of overdiagnosis of allergy. The adoption of 100% sensitivity and 100% specificity cut‐offs is interesting as it allows one to reach total diagnostic accuracy, but it usually leads to a higher number of OFC and a greater proportion of positive OFC, like we have reported before for egg allergy [12]. Applying our findings from this milk allergy study to clinical practice, children with a negative BAT (as defined by the 100% sensitivity cut‐off) can safely introduce BM in their diet, without the need for OFC. Children with a BAT result above the 100% specificity cut‐off are considered BM allergic and could initiate immunomodulatory treatments, such as oral immunotherapy (OIT) with FM, which is more allergenic and therefore more efficacious in terms of desensitization, like has been shown for egg [24]. It is also easier to administer FM for OIT than BM, with the latter, conversely, having a greater risk of adverse events due to the delayed absorption and delayed exposure of allergen epitopes to the immune system. With the recent evidence of greater efficacy of OIT at a younger age and lower allergen‐sIgE at baseline, it is particularly advantageous to test young children and intervene proactively at this early age [25, 26, 27]. The fact that BAT performed particularly well in the younger age group and was not affected by potential immaturity of the immune system or the allergic response, possibly because basophils are part of the innate immune system, makes BAT of particular interest in the management of cow's milk allergy. Infants and young children could have BAT at diagnosis, after they present with suspected milk allergy, to guide proactive management, namely the introduction of BM where possible, without the need for OFC in most cases.

SPT, sIgE, and BAT performed better in BM allergy using BM preparations compared to using cow's milk extracts, suggesting that the food form that is being consumed during oral exposure, namely during OFC, is clinically relevant for testing. All tests, that is, SPT, sIgE, and BAT, had less accurate diagnostic performance for FM allergy compared with BM allergy. The greater discrepancy between the test results and the outcome of FM OFC, compared to BM OFC, is probably related to the longer time gap between tests and OFC to FM (compared to BM OFC) and possibly to the ongoing consumption of BM prior to FM OFC. Despite the 48‐h strict avoidance of cow's milk prior to the FM OFC imposed by the study protocol, which precluded circulating milk proteins, it is possible that the continued exposure prior to that 48‐h period modified the allergic status to FM. We have previously shown an association between the consumption of egg and the diagnostic performance of allergy tests [28]. It is possible that this is also the case for milk. We shall test this hypothesis and look forward to assessing the diagnostic performance of BAT and the other tests for FM allergy in the multicenter BAT Impact study (NCT05309772) where the diagnostic performance of BAT and other tests are secondary endpoints.

In conclusion, in this large rigorous diagnostic study of cow's milk allergy, where all children had OFC, BAT was the best test to identify children who reacted to BM or FM. Using 100% sensitivity and 100% specificity cut‐offs, BAT allowed the lowest need for OFC and 100% diagnostic accuracy.

## Author Contributions

I.B., H.B., R.‐X.F., A.M.‐M., H.F.M., S.R., G.D.T., and A.F.S. performed study procedures related to patient recruitment and cared for study participants. M.K., Z.J., and G.A. performed and analyzed the basophil activation test. F.H. and A.F.S. designed the food frequency questionnaires and oral food challenge protocols. F.H. and M.E. prepared the oral food challenge doses. M.E. and E.P. managed the study data and database. C.R. performed the statistical analyses. A.F.S. designed the study protocol and acted as chief investigator for the study, obtained and managed the research funding, supervised data acquisition, data management and data analyses, and wrote the first version of the manuscript. All authors critically reviewed the manuscript and approved its final version.

## Conflicts of Interest

Dr. Radulovic and Dr. Marshall report salary support from grants from the National Institute of Allergy and Infectious Diseases (NIAID, NIH). Dr. Lack reports grants from the National Institute of Allergy and Infectious Diseases (NIAID, NIH), others from Food Allergy & Research Education (FARE), others from the MRC & Asthma UK Centre, others from the UK Dept of Health through NIHR, others from the National Peanut Board (NPB), others from The Davis Foundation, during the conduct of the study; he is a shareholder in DBV Technologies and Mighty Mission Me, and he receives personal fees from Novartis, personal fees from Sanofi‐Genzyme, personal fees from Regeneron, personal fees from ALK‐Abello, and personal fees from Lurie Children's Hospital, outside the submitted work. Dr Du Toit reports grants from National Institute of Allergy and Infectious Diseases (NIAID, NIH), Food Allergy & Research Education (FARE), MRC & Asthma UK Centre, UK Dept of Health through NIHR, Action Medical Research, and National Peanut Board. Scientific Advisory Board member Aimmune. Investigator on pharma‐sponsored allergy studies (Aimmune, and DBV Technologies). Scientific advisor to Aimmune, DBV, and Novartis. Dr. Santos reports grants from Medical Research Council (MR/M008517/1; MC/PC/18052; MR/T032081/1), Food Allergy Research and Education (FARE), the Immune Tolerance Network/National Institute of Allergy and Infectious Diseases (NIAID, NIH), Asthma UK (AUK‐BC‐2015‐01), BBSRC, Rosetrees Trust, and the NIHR through the Biomedical Research Centre (BRC) award to Guy's and St Thomas' NHS Foundation Trust, during the conduct of the study; personal fees from Thermo Scientific, Novartis, Allergy Therapeutics, DBV, Sanofi/Regeneron and Parexel as well as research support from Buhlmann and Thermo Fisher Scientific through a collaboration agreement with King's College London. The other authors declare no conflicts of interest.

## Supporting information


**Data S1:** all16675‐sup‐0001‐Supinfo.docx.

## Data Availability

The data that support the findings of this study are available on request from the corresponding author. The data are not publicly available due to privacy or ethical restrictions.
