# Supplementary material for: The Basophil Activation Test Is the Most Accurate Test in Predicting Allergic Reactions to Baked and Fresh Cow's Milk During Oral Food Challenges
Source: Allergy. 2025 Aug 13;80(10):2861–73. doi: 10.1111/all.16675 (PMC12486342; doi:10.1111/all.16675)

**Supplementary material**

**The basophil activation test is the most accurate test in predicting allergic reactions to baked and fresh cow’s milk during oral food challenges**

**Short title**: Diagnostic markers of cow’s milk allergy

Irene Bartha*, Holly Boyd*, Ru-Xin Foong*, Marta Krawiec*, Andreina Marques-Mejias*, Hannah F. Marshall*, Suzana Radulovic*, Faye Harrison, Grammatiki Antoneria, Zainab Jama, Matthew Kwok, Ewa Pietraszewicz, Malak Eghleilib, Cristian Ricci, Tom Marrs, Gideon Lack, George Du Toit, Alexandra F. Santos

**shared first-authors*

^1^Department of Women and Children’s Health (Pediatric Allergy), School of Life Course Sciences, Faculty of Life Sciences and Medicine, King’s College London, London, United Kingdom

^2^Peter Gorer Department of Immunobiology, School of Immunology and Microbial Sciences, King’s College London, London, United Kingdom

^3^Children’s Allergy Service, Evelina London Children’s Hospital, Guy’s and St Thomas’ Hospital, London, United Kingdom

^4^Africa Unit for Transdisciplinary Health Research (AUTHeR), North-West University, Potchefstroom, South Africa

***Corresponding Author:**

Alexandra F. Santos

Address: Department of Paediatric Allergy, 2^nd^ floor, South Wing, St Thomas’ Hospital, SE1 7EH London, United Kingdom

Telephone number: +44 (0) 20 7188 0610

Email address: alexandra.santos@kcl.ac.uk

**Table E1.** Dose regimens in grams of protein for oral food challenges.

DBPCFC, double-blind placebo-controlled food challenge.

1. **Baked milk challenges.** *The initial doses will be given only in patients considered to be high-risk (HR). **Cumulative dose does not include the High-Risk doses

| Age | | 6-12 months | 1-3 years | 4-10 years | 11-16 years |
| --- | --- | --- | --- | --- | --- |
| Food | | BAKED COW’S MILK | | | |
| Type of challenge | | Open | DBPCFC | DBPCFC | DBPCFC |
| Number of placebo doses randomly interspersed | | 0 | 1 | 1 | 1 |
| Active doses  (g) | High Risk Dose 1* | 0.003 | 0.003 | 0.003 | 0.003 |
|  | High Risk Dose 2* | 0.01 | 0.01 | 0.01 | 0.01 |
|  | Dose 1 | 0.03 | 0.03 | 0.03 | 0.03 |
|  | Dose 2 | 0.1 | 0.1 | 0.1 | 0.1 |
|  | Dose 3 | 0.2 | 0.3 | 0.3 | 0.3 |
|  | Dose 4 | 0.4 | 0.6 | 0.6 | 0.6 |
|  | Open dose | 0.8 | 1.0 | 1.5 | 2.5 |
|  | **Cumulative protein dose**** | **1.53** | **2.03** | **2.53** | **3.53** |
|  | Total number of muffin/cookies | 1.0 | 1.3 | 1.6 | 2.2 |

**B. Fresh milk challenges.** Dose regimens in grams of protein for DBPCFC to fresh milk according to the different age groups.

*The initial doses will be given only in patients considered to be high-risk (HR). **Cumulative dose does not include the High-Risk doses.

| Age | | 6-12 months | 1-3 years | 4-10 years | 11-16 years |
| --- | --- | --- | --- | --- | --- |
| Food | | FRESH COW’S MILK | | | |
| Type of challenge | | Open | DBPCFC | DBPCFC | DBPCFC |
| Number of placebo doses randomly interspersed | | 0 | 1 | 1 | 1 |
| Active doses  (g) | High Risk Dose 1* | 0.003 | 0.003 | 0.003 | 0.003 |
|  | High Risk Dose 2* | 0.01 | 0.01 | 0.01 | 0.01 |
|  | Dose 1 | 0.03 | 0.03 | 0.03 | 0.03 |
|  | Dose 2 | 0.1 | 0.1 | 0.1 | 0.1 |
|  | Dose 3 | 0.3 | 0.3 | 0.3 | 0.3 |
|  | Dose 4 | 1.0 | 1.0 | 1.0 | 1.0 |
|  | Open dose | 2.5 | 4.0 | 5.0 | 5.5 |
|  | **Cumulative protein dose**** | **3.93** | **5.43** | **6.43** | **6.93** |
|  | Total Milk (g) | 116g | 160g | 189g | 204g |

**Table E2.** Proportion of participants fulfilling each inclusion criterion and their outcomes for the oral food challenge to baked milk. N, number; BM, baked milk; OFC, oral food challenge; SPT, skin prick test.

| Inclusion criteria | N (%) | BM OFC outcome | |
| --- | --- | --- | --- |
| History of an immediate-type allergic reaction to cow’s milk | 125 (83%) | 19 (15%) | POSITIVE |
|  |  | 104 (84%) | NEGATIVE |
| No history of cow’s milk consumption | 28 (19%) | 4 (14%) | POSITIVE |
|  |  | 24 (86%) | NEGATIVE |
| Evidence of IgE sensitisation as documented by SPT and/or serum sIgE | 144 (96%) | 22 (15%) | POSITIVE |
|  |  | 127 (84%) | NEGATIVE |

**Table E3.** Clinical characteristics of positive oral food challenges (OFC) to baked milk (BM) and fresh milk (FM) according to the Practall guidelines. Number of patients and proportion of positive challenges are represented.

| **Symptoms** | **Positive OFC to BM (n=22)** | **Positive OFC to FM (n=71)** |
| --- | --- | --- |
| Skin rash | 7 (32%) | 8 (11%) |
| Skin pruritus | 10 (46%) | 17 (24%) |
| Urticaria and angioedema | 17 (77%) | 30 (42%) |
| Sneezing / itchy nose | 10 (46%) | 17 (24%) |
| Wheezing | 3 (14%) | 2 (3%) |
| Laryngeal symptoms | 11 (50%) | 5 (7%) |
| Subjective gastrointestinal symptoms | 4 (18%) | 11 (16%) |
| Objective gastrointestinal symptoms | 2 (9%) | 5 (7%) |
| Cardiovascular / neurological | 2 (9%) | 1 (1%) |

**Table E4.** Comparison of demographic, clinical and immunological characteristics between milk tolerant children with positive versus negative basophil activation test.

| **Patient characteristics** | **Positive BAT to BM (SI CD203c >=1.88) N=13** | **Negative BAT to BM (SI CD203c <1.88) N=97** | **P value** | **Positive BAT to FM (SI CD203c >=1.29) N=15** | **Negative BAT to FM (SI CD203c <1.29) N=47** | **P value** |
| --- | --- | --- | --- | --- | --- | --- |
| Age (years) | 8 (4; 12) | 4 (2; 7) | **0.004** | 5 (3; 13) | 4 (3; 7) | 0.227 |
| Gender (% females) | 3 (23%) | 46 (47%) | 0.138 | 9 (60%) | 17 (36%) | 0.137 |
| Ethnicity |  |  | 0.777 |  |  | 0.742 |
| - White | 1. (54%) | 64 (57%) |  | 9 (60%) | 26 (55%) |  |
| - Black | 1 (8%) | 9 (9%) |  | 2 (13%) | 7 (15%) |  |
| - Asian | 0 (0%) | 4 (4%) |  | 0 (0%) | 0 (0%) |  |
| - Chinese | 1 (8%) | 6 (6%) |  | 0 (0%) | 2 (4%) |  |
| - Mixed | 4 (31%) | 20 (18%) |  | 4 (27%) | 9 (19%) |  |
| - Other | 0 (0%) | 4 (4%) |  | 0 (0%) | 3 (6%) |  |
| Atopic eczema (%) | 11 (85%) | 75 (77%) | 0.730 | 15 (100%) | 38 (81%) | 0.098 |
| Other food allergies (%) | 13 (100%) | 92 (95%) | 0.512 | 15 (100%) | 41 (87%) | 0.346 |
| Allergic rhinitis (%) | 5 (39%) | 33 (34%) | 0.763 | 5 (33%) | 18 (38%) | 1.0 |
| Asthma (%) | 6 (46%) | 16 (17%) | **0.022** | 4 (27%) | 6 (13%) | 0.237 |
| SPT to cow’s milk extract (mm) | 4 (2; 5) | 3 (0; 4) | **0.023** | 3 (1; 3) | 1 (0; 2) | **0.010** |
| SPT to fresh milk (mm) | 8 (6; 11) | 5 (2; 7) | **0.007** | 4 (4; 6) | 3 (0; 5) | **0.011** |
| SPT to baked milk slurry or milkshake (mm) | 3 (1; 6) | 1 (0; 2) | **0.001** | 5 (4; 6) | 2 (0; 4) | **<0.001** |
| Specific IgE to Boiled Milk (kU_A_/L) | 2.39 (0.93; 10.8) | 0.26 (0.06; 0.81) | **<0.001** | 0.20 (0.04; 1.27) | 0.15 (0.03; 0.46) | 0.312 |
| Specific IgE to Cow’s milk (kU_A_/L) | 3.37 (0.76; 13.75) | 0.43 (0.15; 1.22) | **0.001** | 0.40 (0.12; 3.55) | 0.26 (0.13; 0.70) | 0.230 |
| Specific IgE to Bos d 4 (kU_A_/L) | 0.33 (0.09; 7.02) | 0.10 (0.02; 0.66) | **0.032** | 0.13 (0.02; 0.95) | 0.06 (0.01; 0.16) | 0.169 |
| Specific IgE to Bos d 5 (kU_A_/L) | 0.35 (0.11; 0.86) | 0.15 (0.05; 0.56) | 0.211 | 0.17 (0.12; 0.46) | 0.34 (0.09; 0.34) | 0.072 |
| Specific IgE to Bos d 8 (kU_A_/L) | 2.5 (0.84; 7.65) | 0.14 (0.03;4: 0.46) | **<0.001** | 0.04 (0.02; 1.37) | 0.11 (0.01; 0.26) | 0.639 |

**Figure E1.** Proportion of negative oral food challenges to baked milk (blue) and fresh milk (orange) in the different age groups and the overall cohort.


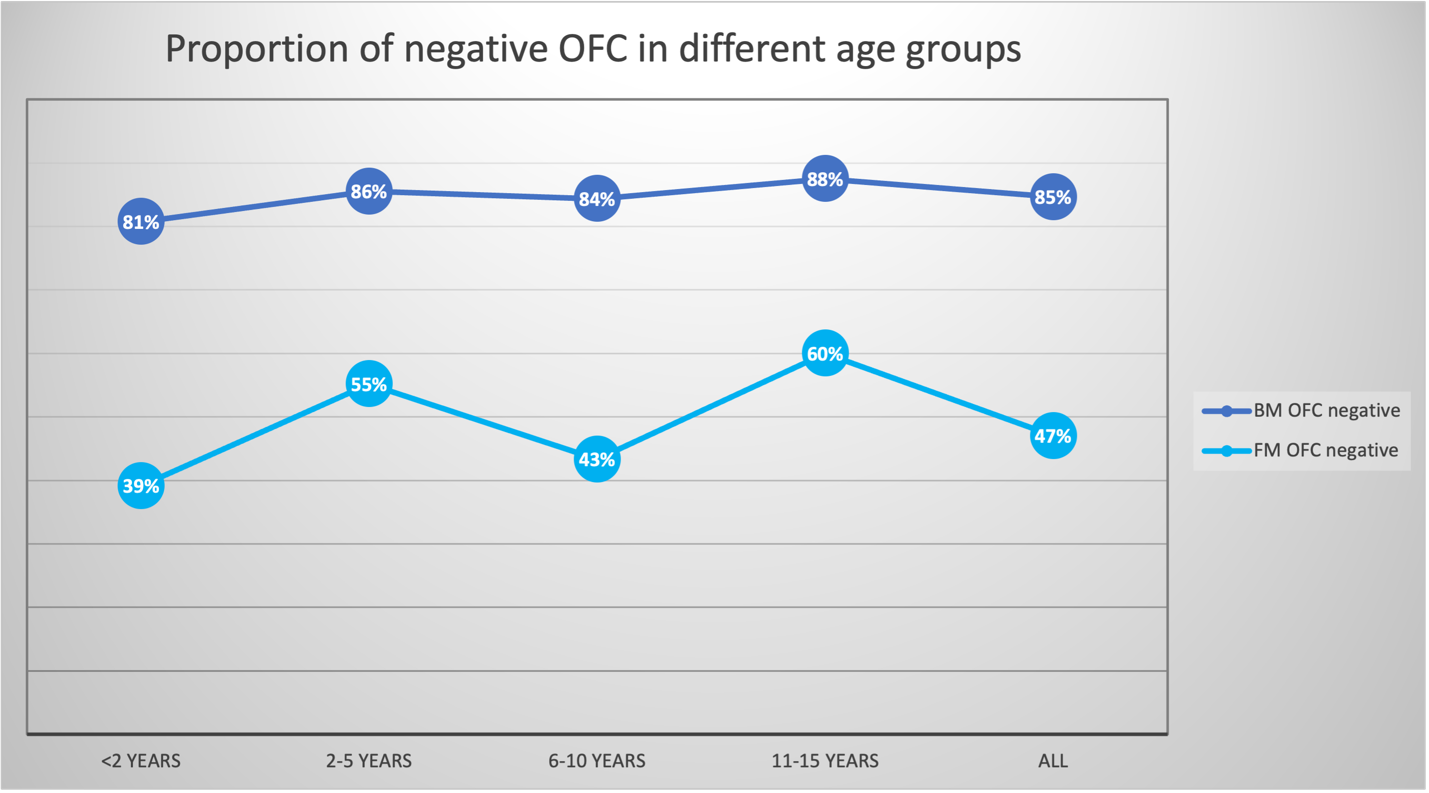


**Figure E2.** Basophil activation test to baked milk and to cow’s milk extract in children with different phenotypes of cow’s milk allergy: allergic to both baked and fresh cow’s milk; allergic to fresh cow’s milk and tolerant to baked milk; and tolerant to both baked and fresh cow’s milk.

#### **Figure E3.** Receiver Operator Characteristic curves for tests using different allergen preparations to predict the outcome of oral food challenges to baked milk and fresh milk.

#### **A.** Skin prick test (SPT) for baked milk allergy – using baked milk (purple), milk extract (turquoise) and fresh milk (blue).

####
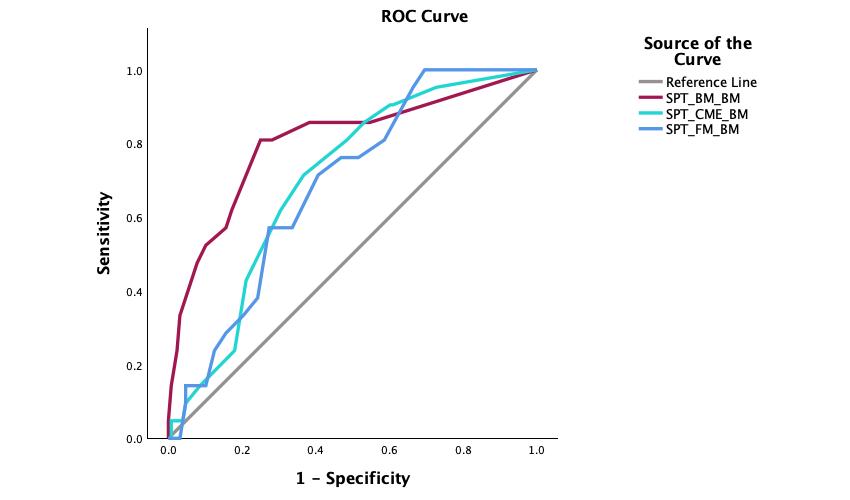


#### **B.** Specific immunoglobulin E for baked milk allergy – to boiled milk (blue), milk extract (green), Bos d 4 (purple), Bos d 5 (turquoise) and Bos d 8 (red).

####
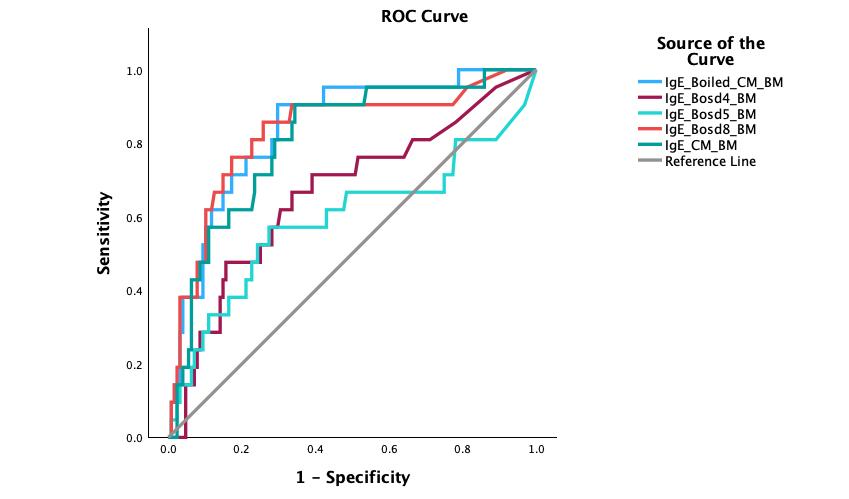


#### **C.** Skin prick test (SPT) for fresh milk allergy – using milk extract (turquoise), fresh milk (red), milkshake used for oral food challenge (green) and difference between SPT to fresh milk and SPT to milk extract.

####
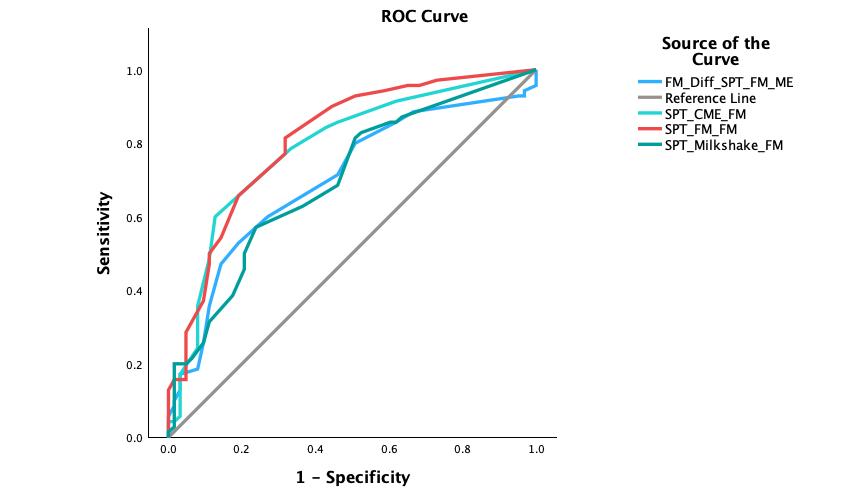


#### **D.** Specific immunoglobulin E for fresh milk allergy – to boiled milk (red), milk extract (green), Bos d 4 (purple), Bos d 5 (turquoise) and Bos d 8 (blue).

####
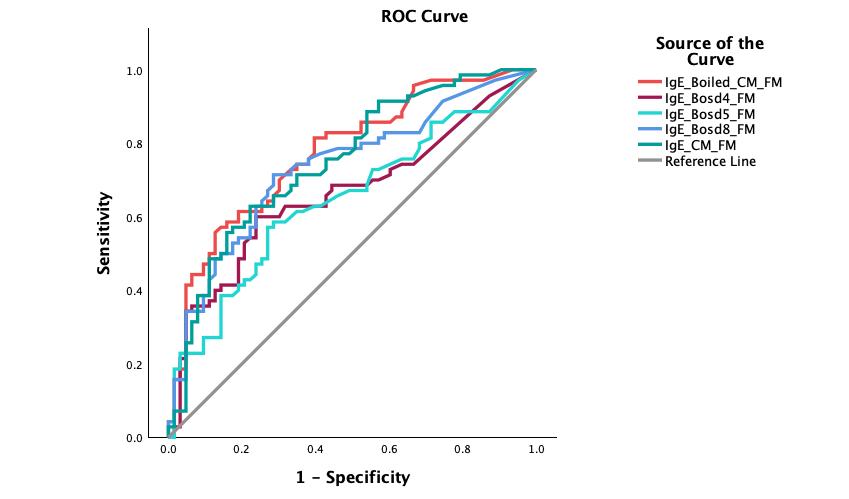


**Figure E4.** Basophil activation and IgE levels at different ages of children with different phenotypes of cow’s milk allergy. Size of the dots relate the IgE levels; allergic in red and tolerant in blue.

1. Baked milk allergy


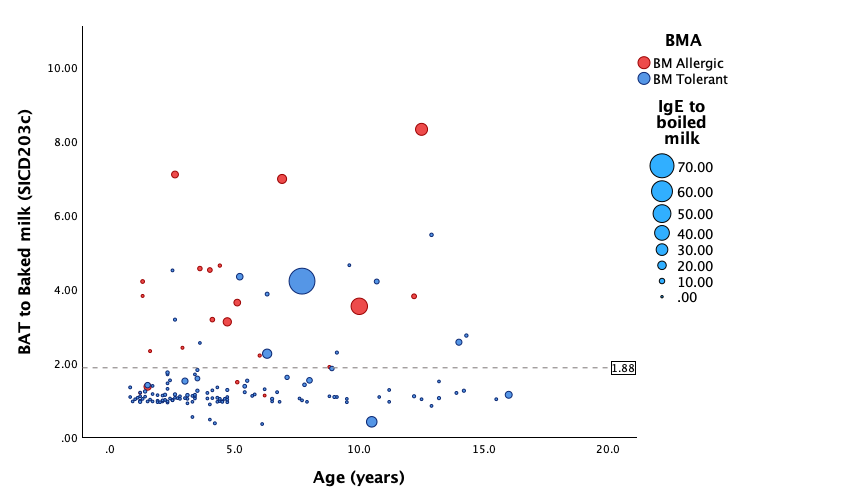


1. Fresh milk allergy


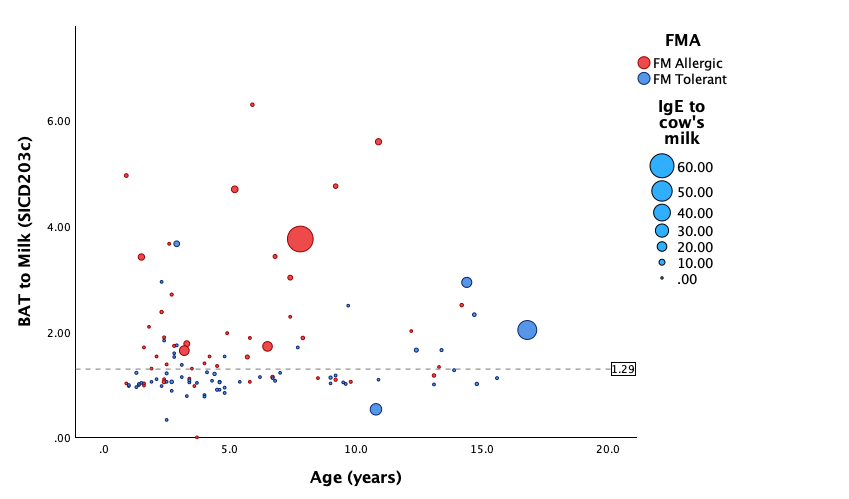

Supplement: Supplementary file 1 — Data S1: all16675‐sup‐0001‐Supinfo.docx. [file ALL-80-2861-s001.docx]
